# Men with HIV Have Increased Alveolar Bone Loss

**DOI:** 10.21203/rs.3.rs-4314428/v1

**Published:** 2024-05-20

**Authors:** Michelle Skelton, Cameron Callahan, Michael Levit, Taylor Finn, Karolina Kister, Satoko Matsumura, Anyelina Cantos, Jayesh Shah, Sunil Wadhwa, Michael Yin

**Affiliations:** Columbia University; Columbia University; Columbia University; Columbia University; Columbia University; Columbia University; Columbia University; Columbia University; Columbia University; Columbia University

**Keywords:** HIV/AIDS, bone biology, men’s health, periodontal disease

## Abstract

**Background:**

Periodontal health in men with HIV remains understudied, despite suggestions of associations between HIV infection and gingival pocketing, periodontal attachment loss, and gingival inflammation. As antiretroviral therapy (ART) has improved the quality of life for people living with HIV (PLWH), aging-related risk factors and comorbidities, including periodontitis, have emerged. This study aims to assess alveolar bone height, gingival crevicular fluid (GCF) cytokines, and periodontal disease activity in men with and without HIV.

**Methods:**

Ninety-three men (50 HIV+, 43 HIV‒) aged 35–70 years were recruited from Columbia University Irving Medical Center clinics. Periodontal examination, GCF collection, and intraoral radiographs were conducted.

**Results:**

While no significant differences were observed in bleeding on probing, clinical attachment loss and pocket depths, men with HIV exhibited significantly greater alveolar crestal height on radiographs compared to men without HIV (HIV + 3.41+/−1.35 mm, HIV− 2.64+/−1.01 mm; p = 0.004), reflecting greater alveolar bone loss. GCF IL6 levels showed a trend towards elevation in men with HIV (HIV + 0.349+/−0.407 pg/ml, HIV− 0.220+/−0.228 pg/ml; p = 0.059).

**Conclusions:**

Men with HIV demonstrate increased alveolar bone loss compared to those without HIV, possibly mediated by elevated IL6 levels. These results underscore the importance of comprehensive oral health management in PLWH and highlight the need for further research understanding the mechanisms linking HIV infection, cytokine dysregulation, and periodontal health.

## Introduction

Within the United States, there are very few current studies investigating the periodontal health in men with HIV. In older studies, it has been suggested that gingival pocketing and destruction of the periodontal attachment are associated with HIV infection [1]. Immunosuppression is suggested to be a risk factor for attachment loss and HIV infection may be a risk factor for gingival inflammation [2]. With the use of effective antiretroviral therapy (ART), people living with HIV (PLWH) experience life expectancies similar to people without HIV and encounter many more aging-related comorbidities [3] than opportunistic infections. A recent study confirmed the protective role of ART as the prevalence of periodontitis in PLWH not receiving ART was two times greater than those PLWH receiving ART [4]. Interestingly, men in the aforementioned study had significantly higher risk of clinical attachment loss than women [4].

Within the general population, men tend to have worsened periodontal disease than women [5]. A recent meta-analysis also concluded that the prevalence of periodontitis was higher in men with HIV (83%) as compared to women with HIV (28%) [6]. In our previous research, we found that postmenopausal women with HIV have lower bone mineral density at the spine and hip compared to postmenopasual women without HIV, accompanied by greater longitudinal bone loss [7, 8]. Additionally, we discovered that while both groups had similar periodontal parameters such as probing depths and clinical attachment loss, postmenopausal women with HIV had significantly more tooth loss (5 fewer teeth) compared to postmenopausal women without HIV [9]. However, the influence of HIV infection on periodontal disease in males still remains unclear.

Prior studies among PLWH have found an association between increased severity of periodontitis and increased prevalence of P. gingivalis, elevated C-reactive protein (CRP) levels, and higher proportions of circulating CD8 + cells [10]. Other studies suggest that ART may have an important role in balancing the salivary microecological oral environment [11]. Consequently, this study seeks to assess alveolar bone loss, gingival crevicular fluid cytokine markers, and periodontal disease activity in men. We hypothesize that the periodontal status of men with HIV will be worse than men without HIV and related to cytokine levels.

## Materials & Methods

### Study Population:

Approval for this study was granted by the Institutional Review Board (IRB-AAA5233) at Columbia University Irving Medical Center. The study adheres to STROBE guidelines for human observational studies. Written informed consent was obtained from all participants involved in this investigation. The research, focusing on the mandibular bone microarchitecture in PLWH, aimed to assess changes in Alveolar Crestal Height (ACH) levels as the primary outcome. From October 2018 to December 2023, we recruited 93 patients, 50 of whom were men with HIV and 43 without HIV, from the dental and Comprehensive Health Program clinics at Columbia University Irving Medical Center.

Inclusion criteria for men with HIV were: a) 35–70 years old; b) physically healthy with no relevant allergies or medical problems; c) HIV-infected as defined by documentation of a positive antibody test or detectable HIV-1 RNA level any time prior to enrollment. In addition, men with HIV had to be on combination ART for at least one year with virological suppression, have a CD4 count > 100 cells/L at time of enrollment, and no opportunistic infections within the last six months prior to enrollment. Inclusion criteria for men without HIV were: a) 35–70 years old; b) physically healthy with no relevant allergies or medical problems; c) a negative HIV antibody test. Exclusion criteria for both groups included: a) history of bisphosphonate or other osteoporosis therapy; b) current testosterone supplementation.

### Periodontal Examination:

A full-mouth periodontal examination was conducted on all participants, recording missing teeth, probing depth (PD), clinical attachment level (CAL), and bleeding on probing (BOP) at six sites per tooth. Periodontal status was classified based on CDC/AAP definitions [12] into: 1) no/mild periodontitis: neither “moderate” nor “severe” periodontitis; 2) moderate periodontitis: ≥2 interproximal sites with CAL ≥ 4 mm (not on same tooth) or ≥ 2 interproximal sites with PD ≥ 5 mm (not on same tooth); 3) severe periodontitis: ≥2 interproximal sites with CAL ≥ 6 mm (not on same tooth) and ≥ 1 interproximal site with PD ≥ 5 mm.

### Gingival Crevicular Fluid (GCF) collection & Inflammatory Cytokine Assays

As described in our previous research [9], gingival crevicular fluid (GCF) samples were collected from the distal site of the maxillary right first molar (#3), the maxillary left central incisor (#9), the maxillary left first premolar (#12), the mandibular left first molar (#19), the mandibular right central incisor (#25), and the mandibular right first premolar (#28). If any of these teeth were absent, the next most anterior tooth in the same quadrant was sampled. After removing supragingival plaque and drying the gingiva, pre-cut periopaper strips (Oraflow, Smithtown, NY, USA) were inserted into the periodontal pocket until mild resistance was felt, ensuring the strip’s midpoint met the distal surface. Strips were held in place for 30 seconds, then placed in a microcentrifuge tube containing 500 L of sterile phosphate buffered saline (PBST; Fisher Scientific Co., Fair Lawn, NJ, USA). After elution by centrifugation, GCF samples were assayed for cytokines (IFN-γ, TNF-a, IL-1β, IL-2, IL-5, IL-6, IL-7, IL-8, IL-10, IL-12p70, IL-13, IL-17A, OPG, and RANKL) in pg/ml and in duplicate at the Salimetrics SalivaLab (Carlsbad, CA), using an electrochemiluminescence method. Calibration curves, generated using a mix of standards, facilitated analyte concentration determination. The average coeficient of variation for all samples tested was <15%. The sample test volume was 25 μL of GCF per determination.

### Intraoral Radiographs:

Participants underwent a full mouth series of intraoral radiographs, taken on the Progeny Preva Unfors-XI (Midmark Corporation, Lincolnshire, Illinois, USA) at 60 kV, 7.0 mA and time range 0.10–0.16 seconds at a 20 cm source-to-skin distance. Similarly to our previous research [9], ACH was measured on posterior bitewing radiographs and anterior periapical radiographs by blinded investigators in up to 24 teeth at mesial and distal sites, excluding third molars and canines.

### Statistical Methods:

Participant demographics and clinical characteristics were summarized, and differences across HIV status were tested. Variation in participant characteristics across HIV status were tested with t-tests for continuous variables and chi-squared tests for categorical variables.

## Results

The rationale of this cross-sectional study was to examine periodontal disease activity in men with and without HIV. A total of 93 men were recruited for the study (50 HIV+, 43 HIV-) with an average age of 52.31+/−8.62 years old (Table 1). Men with HIV had been on cART for an average of 20.47 +/− 7.57 years. There was no significant difference between the HIV status of recruited patients in regards to age, race/ethnicity, smoking status, or diabetes status (Table 1).

### PLWH show similar clinical periodontal variables

There was no significant difference between men with or without HIV on probing depth, bleeding on probing, clinical attachment loss, and number of teeth present (Table 1).

### PLWH have a trend towards increased IL6

GCF levels of IFN-γ, TNF-a, IL-1β, IL-2, IL-5, IL-7, IL-8, IL-10, IL-12p70, IL-13, IL-17 A (pg/ml), OPG, and RANKL were similar in the two groups. GCF IL6 expression was higher in men with HIV (HIV +0.349+/−0.407 pg/ml, HIV− 0.220+/−0.228 pg/ml; p = 0.059) (Table 1).

### PLWH have increased alveolar bone loss

**Figure 1:**
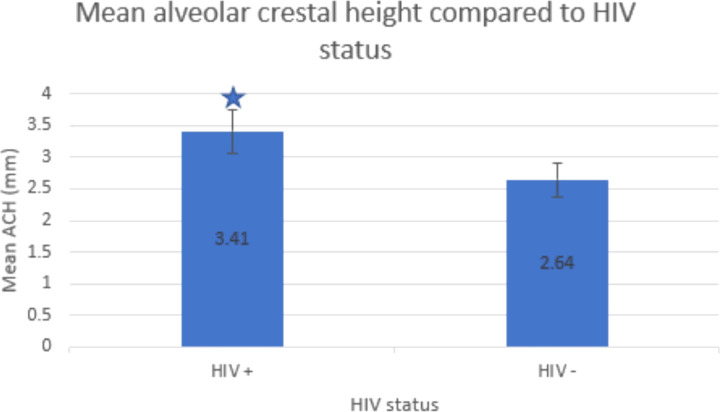
Alveolar crestal height (ACH) in men with and without HIV (n = 93). *significant difference p<0.05 between men with and without HIV

Two-dimensional intraoral radiographs revealed that mean ACH was greater in men with HIV (HIV + 3.41+/−1.35 mm, HIV− 2.64+/−1.01 mm; p = 0.004) than men without HIV. Higher ACH values indicate greater alveolar bone loss (Fig. 1).

## Discussion

In this cross-sectional study of men with and without HIV of similar age, race/ethnicity, smoking and diabetes status, there were no significant differences in the periodontal parameters (probing depth, clinical attachment loss, bleeding on probing, or number of teeth present) or classification. However, there was a difference in alveolar crestal height (ACH), with significantly more alveolar bone loss noted in men with HIV.

A recent study found that particularly in older males with HIV, the severity and prevalence of periodontitis is greater than when compared to controls [13]. However, that study defined severe periodontitis as probing depth ≥ 6mm at any site, whereas our current study defined severe periodontitis as ≥ 2 interproximal sites with CAL ≥ 6 mm (not on same tooth) and ≥ 1 interproximal site with PD ≥ 5 mm. This difference in definition may contribute to the differences in our results.

In our previous research, we found that postmenopausal women with HIV had significantly fewer teeth present on examination (an average of 5 fewer) compared to postmenopausal women without HIV [9]. Our current study did not find a significant difference in the number of teeth between men with and without HIV. One possible explanation to account for this difference is that menopause potentiates alveolar bone loss, causing more teeth to be lost [14]. Another explanation for the difference is that the mean age of the women enrolled in that study (57.04+/−6.25 years old) is higher than the mean age of men enrolled in our current study (52.31+/−8.62 years old) [15]. It is possible that between-group differences in tooth loss would become apparent in an older cohort of men.

We found that there was a trend towards higher GCF IL6 levels in men with HIV compared to those without (p = 0.059). Previous research findings are in agreement that GCF IL6 levels remain higher among men with HIV. It is known that IL6 has demonstrated upregulatory effects, stimulating HIV-1 replication in T-cells and monocyte-derived macrophages [16]. Furthermore, IL6 DNA binding activity has shown to be enhanced by the transactivator of the TAT protein of the human immunodeficiency virus 1 [17, 18], which contributes to the observation of GCF IL6 levels being significantly increased in men with HIV compared to those without HIV. IL6, through its downstream effects, has an influence on the deleterious nature of periodontal disease. It stimulates RANKL, the major cytokine involved in periodontal disease-associated alveolar bone resorption [19], as well as VEGF, which results in increased angiogenesis and vascular permeability, both key features of inflammatory lesions [20].

In a recent study assessing GCF cytokine levels in patients with periodontal disease compared to healthy subjects, researchers found significantly higher levels of IL6 in periodontitis patients, as well as IL8, IL12, and IL17 [21]. Previous research investigating GCF cytokine levels in patients with HIV shows that those with HIV have significantly higher levels of proinflammatory cytokines (IL1, IL6, IL8) and TNF-alpha [22]. While these other cytokines were not found to be significantly higher in our study, these results suggest that IL6 is one of the cytokines that may be relevant in the pathophysiology of periodontitis, and increased IL6 levels seen in HIV infection may contribute to the alveolar bone deterioration seen in PLWH. Therefore, monitoring the microecological environment of cytokines could potentially serve as a diagnostic tool for assessing an individual’s periodontal disease status and susceptibility to the condition.

This study has several limitations. The modest sample size of the study makes it difficult to generalize to other PLWH populations. In addition, this was a cross-sectional study which limits causal inference. Future longitudinal studies are needed to determine whether GCF IL-6 levels are higher among men with HIV and lead to alveolar bone loss, and whether alveolar bone loss leads to tooth loss in older men living with HIV.

## Conclusion

Men with HIV have significantly higher alveolar bone loss than men without HIV. IL6 levels are elevated in men with HIV which may lead to the deterioration of alveolar trabecular bone architecture and contribute to the observed alveolar bone loss. These findings emphasize the importance of comprehensive oral health assessment and management in PLWH, particularly in understanding the underlying mechanisms contributing to periodontal disease progression. Further research is warranted to understand the interplay between HIV infection, cytokine dysregulation, and periodontal health, with the ultimate goal of developing targeted interventions to mitigate oral health disparities in this population.

## Figures and Tables

**Table 1:** Demographics, gingival crevicular fluid (GCF) biomarker cytokine levels, periodontal, and x-ray for the full cohort of men with and without HIV. Biomarkers were performed for Interferon Gamma (IFNγ), Tumor Necrosis Factor Alpha (TNFα), Interleukin (IL) 1 alpha, 2, 5, 6, 7, 8, 10, 12p70, 13, and 17 alpha, Osteoprotegrin (OPG) and Receptor Activator of Nuclear Factor Kappa Beta (RANKL).

Full cohort					
Variable	# Missing	All	HIV −	HIV +	p-value
N		93	43	50	
Age	0	52.31 (8.62)	51.53 (9.24)	52.98 (8.09)	0.6766
Race/Ethnicity	0				0.9917
African American		55 (59.14%)	24 (55.81%)	31 (62.00%)	
Hispanic White		15 (16.13%)	7 (16.28%)	8 (16.00%)	
Hispanic African		3 (3.23%)	2 (4.65%)	1 (2.00%)	
White		20 (21.51%)	10 (23.26%)	10 (20.00%)	
Smoker	2				0.834
No		67 (72.04%)	32 (74.42%)	35 (70.00%)	
Yes		24 (25.81%)	10 (23.26%)	14 (28.00%)	
Diabetes	1				0.1147
No		84 (90.32%)	43 (100.00%)	41 (82.00%)	
Yes		8 (8.60%)	0 (0.00%)	8 (16.00%)	
**Biomarkers (pg/ml)**	**# Not Detectable**				
INF-gamma	39	0.312	0.340	0.288	0.197
TNF-alpha	0	1.140	1.163	1.119	0.432
IL1-beta	0	62.538	68.120	57.194	0.280
IL2	0	0.739	0.783	0.697	0.251
IL5	40	0.038	0.043	0.035	0.091
IL6	21	0.280	0.220	0.349	0.059
IL7	39	0.062	0.071	0.056	0.164
IL8	0	641.355	693.202	591.714	0.304
IL10	2	0.147	0.164	0.131	0.126
IL12p70	26	0.218	0.242	0.194	0.239
IL13	8	3.403	3.549	3.258	0.320
IL17a	27	0.617	0.508	0.722	0.148
OPG	1	5.656	5.313	5.992	0.329
RANKL	28	1.683	1.729	1.636	0.388
**Periodontal variables**	**# Missing**				
Periodontitis	6				0.869
Mild		7 (7.53%)	5 (11.63%)	2 (4.00%)	
Moderate		31 (33.33%)	15 (34.88%)	16 (32.00%)	
Severe		49 (52.69%)	23 (53.49%)	26 (52.00%)	
Mean PD	5	3.49 (1.08)	3.48 (1.15)	3.51 (1.03)	0.911
Mean AL	9	4.12 (1.52)	4.06 (1.38)	4.17 (1.66)	0.737
% BOP	5	0.27 (0.22)	0.24 (0.21)	0.30 (0.23)	0.220
# Teeth Present	2	21.33 (6.62)	22.43 (6.62)	20.39 (6.54)	0.143
Mean ACH			2.64 (1.01)	3.41 (1.35)	0.00374

## Data Availability

All processed data generated or analyzed during this study are included in this published article. The raw datasets generated and/or analyzed during the current study are not publicly available due to protected health information but de-identified data are available from the corresponding author on reasonable request.
